# Energy transfer driven brightening of MoS_2_ by ultrafast polariton relaxation in microcavity MoS_2_/hBN/WS_2_ heterostructures

**DOI:** 10.1038/s41467-024-45554-y

**Published:** 2024-02-26

**Authors:** Zehua Hu, Tanjung Krisnanda, Antonio Fieramosca, Jiaxin Zhao, Qianlu Sun, Yuzhong Chen, Haiyun Liu, Yuan Luo, Rui Su, Junyong Wang, Kenji Watanabe, Takashi Taniguchi, Goki Eda, Xiao Renshaw Wang, Sanjib Ghosh, Kevin Dini, Daniele Sanvitto, Timothy C. H. Liew, Qihua Xiong

**Affiliations:** 1 National Laboratory of Solid State Microstructures, School of Electronic Science and Engineering, and Collaborative Innovation Center of Advanced Microstructures, Nanjing University, Nanjing, 210093 China; 2 Centre for Quantum Technologies, National University of Singapore, Singapore, 117543 Singapore; 3 CNR NANOTEC Institute of Nanotechnology, Lecce, 73100 Italy; 4 Division of Physics and Applied Physics, School of Physical and Mathematical Sciences, Nanyang Technological University, Singapore, 637371 Singapore; 5 Beijing Academy of Quantum Information Sciences, Beijing, 100193 P.R. China; 6 State Key Laboratory of Low-Dimensional Quantum, Department of Physics Physics, Tsinghua University, Beijing, 100084 P.R. China; 7 Department of Physics, National University of Singapore, Singapore, 117542 Singapore; 8 Research Center for Functional Materials, National Institute for Materials Science, 1-1 Namiki, Tsukuba, 305-0044 Japan; 9 International Center for Materials Nanoarchitectonics, National Institute for Materials Science, 1-1 Namiki, Tsukuba, 305-0044 Japan; 10 School of Electrical and Electronic Engineering, Nanyang Technological University, Singapore, 639798 Singapore; 11 Frontier Science Center for Quantum Information, Beijing, 100084 P.R. China; 12 Collaborative Innovation Center of Quantum Matter, Beijing, P.R. China

**Keywords:** Polaritons, Characterization and analytical techniques

## Abstract

Energy transfer is a ubiquitous phenomenon that delivers energy from a blue-shifted emitter to a red-shifted absorber, facilitating wide photonic applications. Two-dimensional (2D) semiconductors provide unique opportunities for exploring novel energy transfer mechanisms in the atomic-scale limit. Herein, we have designed a planar optical microcavity-confined MoS_2_/hBN/WS_2_ heterojunction, which realizes the strong coupling among donor exciton, acceptor exciton, and cavity photon mode. This configuration demonstrates an unconventional energy transfer via polariton relaxation, brightening MoS_2_ with a record-high enhancement factor of ~440, i.e., two-order-of-magnitude higher than the data reported to date. The polariton relaxation features a short characteristic time of ~1.3 ps, resulting from the significantly enhanced intra- and inter-branch exciton-exciton scattering. The polariton relaxation dynamics is associated with Rabi energies in a phase diagram by combining experimental and theoretical results. This study opens a new direction of microcavity 2D semiconductor heterojunctions for high-brightness polaritonic light sources and ultrafast polariton carrier dynamics.

## Abstract (web summary)

Here, the authors design a microcavity-confined 2D heterojunction to realize the strong coupling among donor exciton, acceptor exciton, and cavity photon mode, leading to an unconventional energy transfer v*ia polariton relaxation* with an enhancement factor of *~*440.

## Introduction

Monolayer semiconducting transition metal dichalcogenides (TMDs) have recently emerged as a promising system in fundamental physics and technology-related studies of electronics, optoelectronics, valleytronics, and twistronics. Their prominent properties are enabled by the weak dielectric screening, breaking of the inversion symmetry, and strong spin-orbit coupling^1–4^. Despite being direct bandgap semiconductors, most monolayer TMDs exhibit poor photoluminescence (PL) yield due to the low absolute optical absorption of <10% as well as the low external quantum yield of <1%, which limits the development of nanophotonic devices such as light-emitting diodes (LEDs), lasers, display devices, and optical on-chip networks^5–7^.

One prospective way to achieve a homogeneous enhancement of emission intensity is to deliver the excitation energy from the donor to the acceptor by nonradiative energy transfer. Conventional energy transfer comprises Förster resonance energy transfer (FRET) and Dexter-type energy transfer (DET); the former is based on dipole-dipole coupling (where the dipoles in the semiconductors are excitons), while the latter is based on charge transfer and spin conservation^8–11^. To evaluate the effect of energy transfer, an enhancement factor (*η*) is usually defined as $\eta={I}_{{{{{{\rm{DA}}}}}}}/{I}_{{{{{{\rm{A}}}}}}}$, where *I*_DA_ and *I*_A_ are the PL intensities of the acceptor in the heterojunction and on the bare substrate, respectively. For both FRET and DET in 2D semiconductors, *η* is limited to ~2 to date^8,10,12–14^. High-performance nanophotonic devices call for a new structure or mechanism for energy transfer with a colossal *η* to brighten 2D semiconductors.

Alternatively, for organic-semiconductor heterojunctions with Frenkel excitons, exciton-photon polaritons due to the strong coupling of excitons with microcavity provide a powerful platform to achieve unconventional energy transfer by polariton relaxation^15–20^. In contrast, TMDs feature a more delocalized Wannier-Mott exciton nature^21,22^. Their polaritons inherit the perfect in-plane dipole orientation, strong quantum confinement, and valley polarization, which provides more degree of freedoms to engineer new functionalities in the polariton field. Thus, TMDs polaritons have attracted great attention for the study of Bose-Einstein condensates^23–25^, nonlinear optical processes^26,27^, valley properties^28–30^, LEDs^31^ as well as Moiré heterojunctions^32^. However, it remains elusive to realize the strong coupling of TMDs heterojunctions with microcavity for unconventional energy transfer due to the considerable challenge to avoid the PL quenching by ultrafast charge transfer and meanwhile to achieve the mode match among donor exciton, acceptor exciton, and cavity photon.

Here, by embedding the hBN/MoS_2_/hBN/WS_2_ heterojunction into a Fabry-Pérot (FP) microcavity, we have realized the strong coupling among the donor exciton, acceptor exciton, and cavity photon mode, which leads to the brightening of MoS_2_ with a two-order-of-magnitude enhancement of PL yield (*η* ≈ 440) based on the exciton-photon polariton relaxation. The custom-designed *k*-space transient-reflectivity spectroscopy, which features a better spectral resolution than the real-space measurement for microcavity samples, was applied to evaluate the polariton relaxation dynamics, and a characteristic time as short as ~1.3 ps was extracted. The efficient and ultrafast polariton relaxation is associated with the significantly enhanced intra- and inter-branch exciton-exciton scattering, which overcomes the hot phonon bottleneck effect. Moreover, the phase diagram has been established to correlate the polariton relaxation efficiency with the Rabi energies. This study demonstrates a new branch of microcavity-confined 2D semiconductor heterojunctions with a great potential for ultrafast highly-bright polaritonic light sources.

## Results

### Sample fabrication and optical characterization

**Fig. 1:**
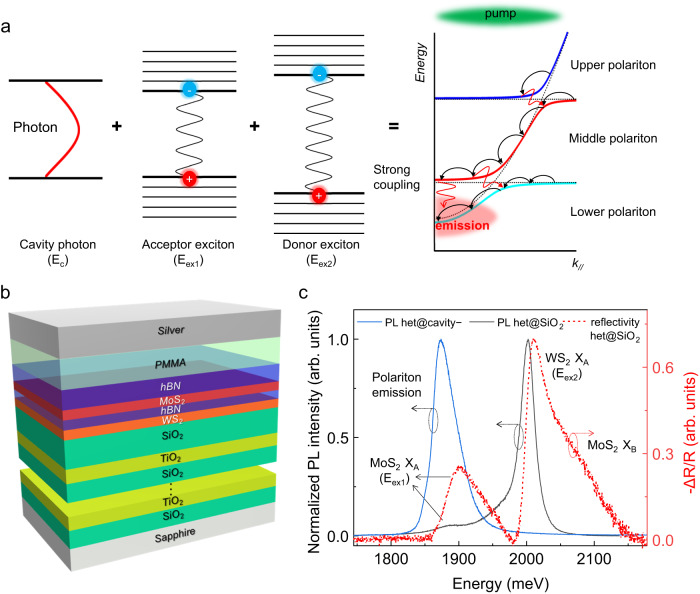
Design, construction, and properties of FP microcavity-confined hBN/MoS_2_/hBN/WS_2_ heterojunction. **a** Schematic formation and relaxation of exciton-photon polariton. The black and red arrows in the right panel represent intra- and inter-branch scattering, respectively. **b** Schematic illustration of microcavity-confined hBN/MoS_2_/hBN/WS_2_ heterojunction. **c** Normalized real-space PL spectra of het@SiO_2_ (black) and het@cavity− (blue). The differential reflection spectrum of het@SiO_2_ (dashed red) is presented for reference. X_A_ and X_B_ are A and B exciton, respectively.

For the optical microcavity-confined heterojunction, the strong coupling among the acceptor’s (E_ex1_) and donor’s (E_ex2_) excitons as well as the cavity photon mode (E_c_) will form the exciton-photon polariton with three anti-crossing eigenstates denoted by the upper (UPB), middle (MPB), and lower (LPB) polariton branches (Fig. 1a)^15–19^. Under off-resonance optical excitation, the polariton tends to relax from the UPB to MPB and finally to LPB (i.e., from high energy and high in-plane momentum (*k*_*//*_) to low energy and low *k*_*//*_). The relaxation process is an unconventional energy transfer since the UPB and LPB are mostly E_ex2_-like and E_ex1_-like, respectively, as determined by the Hopfield coefficients^15–19^.

The configuration of microcavity-confined heterojunction in this study is illustrated in Fig. 1b. The hBN/MoS_2_/hBN/WS_2_ heterojunction was fabricated by a pick-up method (Supplementary Fig. 1)^32^. WS_2_ (donor) and MoS_2_ (acceptor) are monolayers with a direct bandgap, while the top and middle hBN are 15 and 2 nm, respectively. The middle thin hBN layer prevents the ultrafast interlayer charge transfer from nonradiative recombination while keeping the FRET almost intact^13,33,34^. Then, the heterojunction was transferred onto a bottom-distributed Bragg reflector (DBR) composed of 6.5 pairs of SiO_2_ and TiO_2_ dielectric layers, with its photonic stopband covering the heterojunction exciton energies (Supplementary Fig. 2). Subsequently, a poly(methyl methacrylate) (PMMA) spacer and a silver mirror were deposited to finalize the microcavity device. The thickness of PMMA spacer was adjusted to achieve the negative (positive) acceptor exciton-photon detuning, with the microcavity heterojunction labeled as het@cavity− (het@cavity+), respectively. Supplementary Table 1 summarizes the thickness information of het@cavity−, which ensures an energy match between the cavity mode and heterojunction excitons, i.e., the heterojunction placed in the maximum of the electromagnetic field. For quantitative comparison, an identical heterojunction was fabricated on a SiO_2_(90 nm)-coated Si substrate to avoid the cavity effect, defined as het@SiO_2_ (Supplementary Fig. 3a).

Figure 1c compares the normalized real-space PL spectra of het@cavity− and het@SiO_2_, with the differential reflection spectrum of het@SiO_2_ as a reference. The real-space PL spectrum of het@SiO_2_ features two emission peaks, i.e., A exciton (X_A_) emission from WS_2_ at 2002 meV and MoS_2_ at 1890 meV, with a Stokes shift of ~10 meV relative to the corresponding reflectivity spectrum. The PL intensity of WS_2_ X_A_ is much stronger than that of MoS_2_ X_A_, which results from the higher intrinsic exciton dipole oscillator strength (*f*) and quantum yield of the former, and the interlayer FRET (*η* ≈ 3), as detailed in Supplementary Fig. 3. On the contrary, the PL spectrum of het@cavity− is only composed of a single peak at 1873 meV, with the even smaller energy than the PL of MoS_2_ X_A_. The significant change of PL spectra, in both shape and energy, points out the crucial effect of the optical microcavity confinement on energy transfer.

**Fig. 2:**
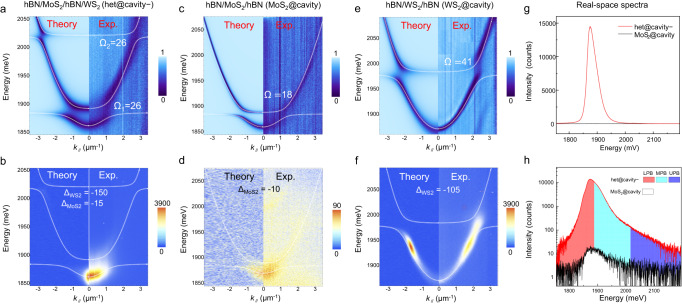
Comparison of polariton relaxations in het@cavity−, MoS_2_@cavity and WS_2_@cavity. **a**, **b** *k*-space energy-resolved reflectivity mapping (**a**), PL mapping (**b**) for het@cavity − . **c**, **d** The corresponding results for MoS_2_@cavity. **e**, **f** The corresponding results for WS_2_@cavity. The Rabi splitting (Ω) and exciton-photon detuning (Δ) energies are labeled in the unit of meV. Note: data range of the color bar in (**d**) is much smaller than that in (**b**) and (**f**). **g**, **h** The real-space PL spectra of het@cavity− and MoS_2_@cavity in linear (**g**), and log (**h**) scale under 10 μW excitation.

The energy transfer in het@cavity− is further understood by means of the *k*-space energy-resolved reflectivity and PL mappings, in comparison with the cases of microcavity-confined hBN/MoS_2_/hBN (MoS_2_@cavity) and hBN/WS_2_/hBN (WS_2_@cavity), as summarized in Fig. 2a–f. The polariton dispersions for het@cavity− are fitted with a three coupled oscillator model^15–17^, *i.e*.,

1 $$\left(\begin{array}{ccc}{E}_{c}\left(\theta \right) & {\Omega }_{2}/2 & {\Omega }_{1}/2\\ {\Omega }_{2}/2 & {E}_{{ex}2} & 0\\ {\Omega }_{1}/2 & 0 & {E}_{{ex}1}\end{array}\right)\left(\begin{array}{c}\begin{array}{c}{\alpha }_{c}\\ {\alpha }_{{ex}2}\end{array}\\ {\alpha }_{{ex}1}\end{array}\right)={E}_{{pol}}\left(\theta \right)\left(\begin{array}{c}\begin{array}{c}{\alpha }_{c}\\ {\alpha }_{{ex}2}\end{array}\\ {\alpha }_{{ex}1}\end{array}\right)$$

where ${{E}_{c}\left(k \right)={E}_{0}(1-{\sin }^{2}(\theta )/{n}^{2})}^{-1/2}$ is the cavity photon mode energy, $\theta$ is the angle of incidence, *E*_0_ is the cavity cut off energy, and *n* is the cavity effective refractive index. *E*_ex1_, *E*_ex2_ and *E*_pol_(*θ*) are the energies of acceptor exciton, donor exciton, and polariton, respectively. Ω_1_ and Ω_2_ are the corresponding Rabi splitting energies. *α*_*ex1*_*, α*_*ex2*_ and *α*_*c*_ are the corresponding Hopfield coefficients (Supplementary Fig. 4). The angle dependence of the three eigenvalues corresponds to three dispersion branches termed UPB, MPB, and LPB, respectively (the white dotted lines in Fig. 2a, b). Similarly, the polariton dispersions for MoS_2_@cavity and WS_2_@cavity are fitted with a two-coupled oscillator model, which gives two branches, termed UPB and LPB (see Method).

The reflectivity mappings of MoS_2_@cavity and WS_2_@cavity show two anti-crossing branches with Rabi splitting energy (Ω) of 18 and 41 meV, respectively (Fig. 2c, e), consistent with the smaller exciton dipole oscillator strength of MoS_2_ than WS_2_ ($\Omega \propto \sqrt{f}$, Supplementary Fig. 3b)^35,36^. In contrast, het@cavity− features three branches with two anti-crossing points, demonstrating the strong coupling among MoS_2_ exciton, WS_2_ exciton, and cavity photon mode, with Ω_1_ (Ω_2_) ≈ 26 (26) meV and a negative (negative) exciton-photon detuning (Δ) of −15 (−150) meV for MoS_2_ (WS_2_) (Fig. 2a). Ω_1_ of het@cavity− is larger than Ω of MoS_2_@cavity and Ω_2_ of het@cavity− is smaller than Ω of WS_2_@cavity, which straightforwardly indicates the transfer of dipole oscillator strength from E_ex2_ to E_ex1_^37^. A similar transfer is also reflected in het@cavity+ with a Ω_1_ (Ω_2_) ≈ 42 (24) meV and a positive (negative) Δ of +13 (−132) meV for MoS_2_ (WS_2_) (Supplementary Fig. 5a). These results suggest a general feature of the transfer of dipole oscillator strength in the strongly coupled microcavity-confined heterojunctions, in sharp contrast to the negligible transfer in the cavity-free het@SiO_2_ counterpart (Supplementary Fig. 3b). The experimental reflectivity mappings are well-reproduced by theoretical calculation with the transfer matrix method (Fig. 2a, c, e, detailed in Supplementary part III)^38–40^.

The PL mapping of MoS_2_@cavity suffers from a weak polariton emission due to the weak dipole oscillator strength and low quantum yield of MoS_2_ (Fig. 2d)^5^, while that of WS_2_@cavity suffers from a hot phonon bottleneck effect (the broad emission centred at ~1.7 μm^−1^) due to the large negative Δ (−105 meV, Fig. 2f)^29,41,42^. On the contrary, the PL mapping of het@cavity− integrates the advantages of MoS_2_@cavity and WS_2_@cavity, *i.e*., inherits the narrow linewidth from MoS_2_@cavity and high intensity from WS_2_@cavity (Fig. 2b); so does the PL mapping of het@cavity+ (Supplementary Fig. 5b). The PL intensity of het@cavity− in real-space photoluminescence excitation spectrum increases monotonously without resonance peak when decreasing the excitation energy towards LPB (Supplementary Fig. 6). The preceding results confirm the energy transfer mechanism of polariton relaxation, rather than FRET or DET, in the strongly coupled microcavity-confined heterojunctions. In the linear-scale real-space PL spectrum, het@cavity− and MoS_2_@cavity show bright and negligible emission under 10 μW excitation, respectively (Fig. 2g). From Fig. 2b, it is learned that the polariton emission of het@cavity− comes from the LPB and MPB with |*k*_*//*_| < 1.6 μm^−1^, which is composed of MoS_2_ exciton and cavity photon but negligible WS_2_ exciton (Supplementary Fig. 4a). Hence, *η* is obtained to be ~440 from the ratio of ${I}_{{het@cavity}-}^{{LPB}+{MPB}}/{I}_{{{MoS}}_{2}{@cavity}}^{{LPB}+{UPB}}$. Here ${I}_{{het@cavity}-}^{{LPB}+{MPB}}$ and ${I}_{{{MoS}}_{2}{@cavity}}^{{LPB}+{UPB}}$ are the integrated polariton emission intensity from LPB + MPB (red+cyan region) of het@cavity− and LPB + UPB of MoS_2_@cavity (black-outlined region) in real-space, respectively (Fig. 2h, detailed in Supplementary Note 1). As summarized in Supplementary Table 2, the giant *η* of 440 for het@cavity− is over 146 times larger than the *η* of 3 with FRET for het@SiO_2_ counterpart, also two orders of magnitude higher than the typical *η* of FRET or DET processes in literature, demonstrating a powerful strategy to significantly increase the energy transfer efficiency by polariton relaxation.

Supplementary Figs. 7 and 8 show the optical characteristics of the additional microcavity-confined heterojunction samples, specifically identified as het8@cavity (8 nm hBN), het2@node@cavity (heterojunction with 2 nm hBN at the node), and het60@cavity (60 nm hBN). These diverse configurations, characterized by different heterojunction’s positions within the cavity and/or different thicknesses of hBN, give rise to distinct Rabi energies Ω_1_ and Ω_2_. Consequently, the distinct Rabi energies lead to a substantial difference in polariton relaxation efficiency. This result demonstrates the crucial role of Rabi energies in polariton-mediated energy transfer, which has not been recognized to date and will be further theoretically clarified later.

### Theoretical simulation

We simulated the *k*-space PL mappings with the coupled rate equations^43–46^, which describe the dynamics of polariton populations in different branches. The different points with the different relative excitonic and photonic fractions in $k$-space for each branch are identified by the coupled oscillator model and the Hopfield coefficients (Supplementary Fig. 4). The coupled rate equations for het@cavity− with three polariton branches read

2 $$\frac{d{N}_{j}^{\Xi }}{{dt}}=\left({P}_{1}{X}_{1,j}^{\Xi }+{P}_{2}{X}_{2,j}^{\Xi }-\gamma {C}_{j}^{\Xi }\right){N}_{j}^{\Xi }+{\sum }_{{\Xi }^{{\prime} }}{S}^{\Xi {\Xi }^{{\prime} }}$$

where ${N}_{j}^{\Xi }$ is the polariton population, $\Xi=\left\{U,M,L\right\}$ corresponds to the UPB, MPB, and LPB, 1 (2) indicates MoS_2_ (WS_2_), and index $j$ denotes the respective point in $k$-space. ${P}_{1(2)}$ stands for an effective pumping for the exciton, and $\gamma$ for the decay rate of the photon mode. Here, ${P}_{2} > {P}_{1}$ due to the much brighter polariton emission of WS_2_@cavity than MoS_2_@cavity (Fig. 2d, f). The ${X}_{1(2)}^{\Xi }$ and ${C}^{\Xi }$ denote ${|{\alpha }_{1(2)}|}^{2}$ and ${\left|{\alpha }_{c}\right|}^{2}$ (Supplementary Fig. 4). The relaxation processes, described by the last term in (2), consist of the intra- and inter-branch transitions originating from the phonon-mediated exciton scattering^47,48^. In this formalism, the preferred transition with a high rate happens between initial and final destinations with high ${X}_{1}^{\Xi }{X}_{1}^{{\Xi }^{{\prime} }},{X}_{2}^{\Xi }{X}_{2}^{{\Xi }^{{\prime} }}$ and phonon population ${n}_{{ph}}({E}_{{ph}})$. According to Bose distribution function, *i.e*., ${n}_{{ph}}=\frac{1}{\exp \left(\frac{{E}_{{ph}}}{{k}_{B}T}\right)-1}$ (here ${E}_{{ph}}=|{E}^{{\Xi }^{{\prime} }}-{E}^{\Xi }|$), ${n}_{{ph}}$ is large for transitions with close energies. The photonic part of the numerical steady-state solution for ${N}_{j}^{\Xi }$, together with an added Gaussian broadening, agrees with our experimental result (Fig. 2b, d, f; Supplementary part III for details).

Specifically, for MoS_2_@cavity, *P*_1_ excites the population in the low- (high-) *k*_*//*_ region of UPB (LPB) with high exciton fractions ${X}_{1}^{U}({X}_{1}^{L})$ (Supplementary Fig. 4b). The population will relax by the inter-branch scattering (from UPB to LPB) due to the high ${X}_{1}^{U}{X}_{1}^{L}$ at the anti-crossing point and the high ${n}_{{ph}}$ (*E*_*ph*_ ≈ Ω ≈ 18 meV), followed by the intra-branch scattering (within LPB) due to the non-negligible ${{X}_{1}^{L}X}_{1}^{{L}^{{\prime} }}$ and the high ${n}_{{ph}}$ (flat dispersion)^47,48^. However, the polariton emission is considerably weak due to the weak oscillator strength and low quantum yield of MoS_2_ exciton (Supplementary Fig. 3). For WS_2_@cavity, *P*_2_ also excites the population in the low- (high-) *k*_*//*_ region of UPB (LPB) with high exciton fractions ${X}_{2}^{U}$ (${X}_{2}^{L}$) (Supplementary Fig. 4c). However, only a tiny portion of the population will relax to the bottom of LPB due to the poor inter-branch scattering for the low ${n}_{{ph}}$ (*E*_*ph*_ ≈ Ω ≈ 41 meV) and the poor intra-branch scattering for the negligible ${{X}_{2}^{L}X}_{2}^{{L}^{{\prime} }}$, presenting the troublesome hot phonon bottleneck (Fig. 2f)^29,41^.

Based on the above experimental and theoretical results, the polariton relaxation in het@cavity− could be well elucidated (Fig. 2b). Due to ${P}_{2}\, > \,{P}_{1}$, *P*_2_ excites the most population in the *k*_*//*_ < 2.5 (>2.5) μm^−1^ region of UPB (MPB) with high ${X}_{2}^{U}$ (${X}_{2}^{M}$) (Supplementary Fig. 4a). The population will relax by the inter-branch scattering (from UPB to MPB) due to the high ${X}_{2}^{U}{X}_{2}^{M}$ at the top anti-crossing point and the high ${n}_{{ph}}$ (*E*_*ph*_ ≈ Ω_2_ ≈ 26 meV). The subsequent intra-branch scattering within MPB is faster than that within LPB for WS_2_@cavity due to the non-negligible ${{X}_{2}^{M}X}_{2}^{{M}^{{\prime} }}$ and ${{X}_{1}^{M}X}_{1}^{{M}^{{\prime} }}$ for the former compared to the negligible ${{X}_{2}^{L}X}_{2}^{{L}^{{\prime} }}$ for the latter. The synergism of the two scatterings leads to a fast relaxation of almost all the population to the bottom anti-crossing point. The population will further relax towards the bottom of LPB in a way similar to that in MoS_2_@cavity, as reflected in Fig. 1a. Such a fast polariton relaxation dynamics leads to the bright PL of het@cavity− at the bottom of LPB (Fig. 2b).

### Dynamics and phase diagram

**Fig. 3:**
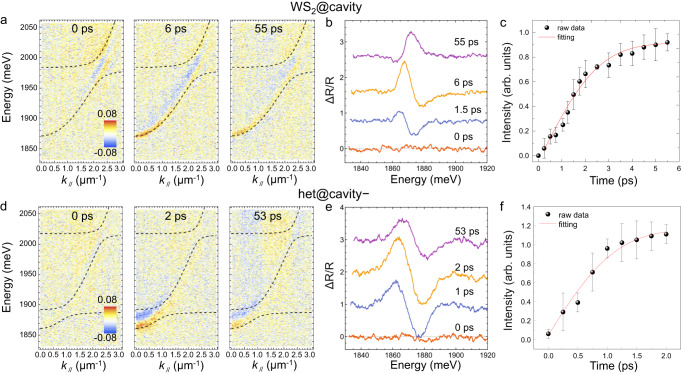
Time-resolved polariton relaxation dynamics. **a**–**c** *k*-space transient-reflectivity spectroscopy mapping (**a**), the integrated $\triangle R/R$ in 0 ~ 0.33 μm^−1^ at typical time delays (**b**), and the intensity evolution of the derivative signal (**c**) of WS_2_@cavity. **d**–**f** The corresponding data of het@cavity−. Note: The steady-state polariton branches are shown in dashed black to guide the reading. The error bars in (**c**) and (**f**) correspond to the standard error of the data points.

With a custom-designed microscopic *k*-space transient-reflectivity spectroscopy (see Methods), we have simultaneously unravelled the evolution of the polariton population in momentum, energy and time domains. After being pumped by a 200-fs laser pulse of ~2 μW at 580 nm, the change of reflectivity $(\frac{\triangle R}{R}=\frac{{R}_{{{{{{\rm{pump\; on}}}}}}}-{R}_{{{{{{\rm{pump\; off}}}}}}}}{{{{{{{\rm{R}}}}}}}_{{{{{{\rm{pump\; off}}}}}}}})$ of the broadband white light is measured after a specific time delay, where ${R}_{{{{{{\rm{pump\; on}}}}}}}$ and ${R}_{{{{{{\rm{pump\; off}}}}}}}$ are the reflectivity mappings with or without optical pumping, respectively. Such an excitation fluence is low enough to avoid exciton-exciton annihilation^49,50^. The *k*-space transient-reflectivity spectroscopy results of WS_2_@cavity and het@cavity− are shown in Fig. 3, with the corresponding movies in Supplementary Material. For the WS_2_@cavity, the laser pulse excites the exciton reservoir to quickly form polaritons, which induces a net photobleaching signal at 0 ps at *k*_*//*_ ≈ 2.3 μm^−1^ (Fig. 3a), also demonstrated in the integrated $\triangle R/R$ at *k*_*//*_ from 2.1 to 2.7 μm^−1^ (Supplementary Fig. 9). Then, a derivative signal emerges at *k*_*//*_ ≈ 0 μm^−1^ region, as clarified by the integrated $\triangle R/R$ at *k*_*//*_ = 0 ~ 0.33 μm^−1^ (Fig. 3a, b and the movie in Supplementary Material). Such a signal represents the blue-shift of LPB due to the polariton-polariton repulsive interaction or the phase-space-filling effect of exciton (Supplementary Figs. 10, 11)^51^. The signal intensity gradually increases, reaches the maximum at ~6 ps, and slowly decays afterward (Fig. 3a). By fitting the evolution of the derivative signal with the rising function (*y* = A + B erf (*t*/*τ*), where ${{{{{\rm{erf}}}}}}(z)=\frac{2}{\sqrt{\pi }}{\int }_{0}^{z}{e}^{-{t}^{2}}{dt}$), a characteristic rising time (*τ*) of ~2.8 ± 0.4 ps is obtained (Fig. 3c)^52^. In contrast, for het@cavity−, the intensity of the derivative signal at *k*_*//*_ ≈ 0 μm^−1^ region increases much faster and reaches the maximum at ~2 ps (Fig. 3d, e). With the same fitting method, a much shorter *τ* of ~1.3 ± 0.2 ps is obtained (Fig. 3f), which approximates to the *τ* of 1.2 ± 0.2 ps in MoS_2_@cavity (Supplementary Fig. 12). Therefore, het@cavity− demonstrates the ultrafast energy transfer processes accompanied by the highest enhancement factor (440) to date (Supplementary Table 2).

**Fig. 4:**
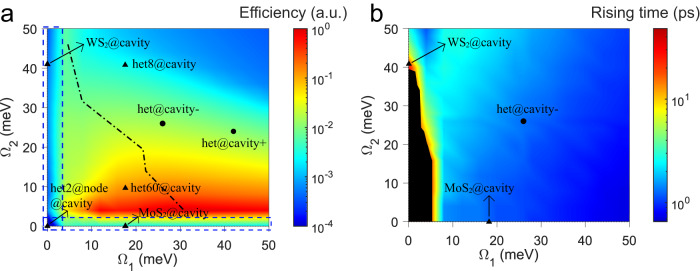
Phase diagram of the polariton relaxation dynamics versus Rabi energies. **a**, **b** Polariton relaxation efficiency (**a**) and characteristic rising time (**b**) with respect to Rabi energies Ω_1_ and Ω_2_. The seven specific experimental cases are marked. In **a**, the dashed-dotted curve indicates the optimal efficiency concerning ${\Omega }_{1}$ and ${\Omega }_{2}$, and the dashed blue boxes show regions where ${\Omega }_{1},{\Omega }_{2}\to 0$. Note: In the dark area of **b**, the population in the low-*k*_*//*_ region of LPB does not rise initially.

Based on the preceding qualitative and quantitative analysis, we have formulated the phase diagram that correlates the polariton relaxation efficiency and characteristic rising time with the Rabi energies Ω_1_ and Ω_2_, as shown in Fig. 4 (see Supplementary part III for details). Here we define a measure of efficiency as the sum of the photonic part of LPB population in the steady-state, *i.e*., ${{{{{\mathscr{N}}}}}}\left({\tau }_{s}\right)\equiv \sum {N}_{j}^{L}\left({\tau }_{s}\right){C}_{j}^{L}$ only for low-*k*_//_ points (${\tau }_{s}$ denotes the time at steady state). In general, ${{{{{\mathscr{N}}}}}}\left({\tau }_{s}\right)$ monotonously increases with decreasing ${\Omega }_{2}$ (except for ${\Omega }_{2}\to 0$) due to the enhanced interbranch scattering between UPB and MPB. For a specific ${\Omega }_{2}$, ${{{{{\mathscr{N}}}}}}\left({\tau }_{s}\right)$ depends on the trade-off between the intra-branch scattering in MPB and the inter-branch scattering (MPB to LPB), hence a suitable ${\Omega }_{1}$ is needed for the optimal ${{{{{\mathscr{N}}}}}}\left({\tau }_{s}\right)$ as marked by the dashed-dotted curve (Fig. 4a). For the ${{{{{\mathscr{N}}}}}}\left({\tau }_{s}\right)$ of specific cases, the het@cavity − , het@cavity+ and het60@cavity feature high efficiency. In contrast, ${{{{{\mathscr{N}}}}}}\left({\tau }_{s}\right)$ is low for ${\Omega }_{2}\to 0$ or ${\Omega }_{1}\to 0$ (such as MoS_2_@cavity, WS_2_@cavity, and het2@node@cavity), or Ω_1_ and Ω_2_ are both large (such as het8@cavity), in agreement with the experimental results. Such an agreement offers a deep insight into the polariton-mediated energy transfer, which is inherently dominated by the Rabi energies.

Different from the ${{{{{\mathscr{N}}}}}}\left({\tau }_{s}\right)$, the characteristic rising time is closely related to the scattering rate rather than the final polariton population in the low-*k*_*//*_ region of LPB (Fig. 4b). After a Gaussian effective pump with amplitudes $\{{P}_{1},{P}_{2}\}/50\gamma$, the evolution of the photonic part of the LPB population, *i.e*., ${{{{{\mathscr{N}}}}}}\left(t\right)\equiv \sum {N}_{j}^{L}\left(t\right){C}_{j}^{L}$, is fitted by the rising function. For (${\Omega }_{1},{\Omega }_{2}$) located in the dark region, the negligible polariton fraction in the low-*k*_*//*_ region of LPB prevents ${{{{{\mathscr{N}}}}}}\left(t\right)$ from growing. For ${\Omega }_{1}\gtrsim 8$ meV, the rising time monotonously decreases with increasing ${\Omega }_{1}$, which can be attributed to the dominant intra-branch scattering for the flatter LPB dispersion. Although the larger ${\Omega }_{1}$ means the less transition from MPB to LPB, once the population relaxes to LPB, the strong intra-branch scattering leads to the faster relaxation to low-$k$ points. It is worth noting that the faster rising time might not end up with a higher ${{{{{\mathscr{N}}}}}}\left({\tau }_{s}\right)$, as evidenced by the comparison in Fig. 4a and Fig. 4b. The dependence of the rising time on ${\Omega }_{2}$ is associated with the supply of population that will later end up in the low-*k*_*//*_ region of LPB. Smaller ${\Omega }_{2}$ results in more population relaxation from UPB to MPB, which allows faster growth of ${{{{{\mathscr{N}}}}}}\left(t\right)$, as supported by the longer rising time of WS_2_@cavity than het@cavity− and MoS_2_@cavity (Fig. 4b), in agreement with the experimental result (Fig. 3).

## Discussion

In summary, we have successfully designed and constructed a planar optical microcavity-confined MoS_2_/hBN/WS_2_ heterojunction, in which the middle insulating hBN prevents the charge transfer-induced PL quenching. Such a configuration realizes the strong coupling among donor exciton, acceptor exciton and cavity photon mode, which results in the unconventional energy transfer mechanism of polariton relaxation in 2D material heterojunctions for the first time. Consequently, we have brightened the MoS_2_ with the record-high energy transfer enhancement factor of ~440, which is two-order-of-magnitude higher than the data reported to date. A short characteristic time of ~1.3 ps is extracted for the ultrafast polariton relaxation dynamics, resulting from the significantly enhanced intra- and inter-branch exciton-exciton scattering to overcome the hot phonon bottleneck effect. The formulated phase diagram in this study establishes the correlation between the polariton relaxation dynamics and Rabi energies, which deepens the understanding on the underlying physics. This study drives the collaborative development of energy transfer and polariton fields toward the new topics of dark exciton energy transfer, valley-polarized energy transfer, and high-brightness ultrafast polaritonic light sources.

## Methods

### Sample fabrication

The bottom DBR was deposited on a sapphire substrate by using an e-beam evaporator (Cello, Ohmiker-50B), and was composed of 6.5 alternating pairs of titanium dioxide (TiO_2_, *n* = 2.498) and silicon dioxide (SiO_2_, *n* = 1.478). The hBN/MoS_2_/hBN/WS_2_ heterojunctions were fabricated using a dry-transfer method with a polypropylene carbonate (PPC) stamp. Monolayer WS_2_, monolayer MoS_2,_ and thin hBN flakes were exfoliated onto silicon substrates with a 90-nm SiO_2_ layer. A PPC stamp was used to pick up the flakes in sequence by a home-built micro-transfer stage (Supplementary Fig. 1). Then, the heterojunction was released to the DBR substrate, immersed in acetone overnight to remove the PPC, and annealed in a high vacuum (<10^−6^ mbar) at 200 °C for 2 hours. The top PMMA layer was spin-coated and the top silver mirror was deposited by a thermal evaporator.

### Two coupled oscillator model

Similar to het@cavity−, the polariton dispersion in MoS_2_@cavity and WS_2_@cavity can be fitted with two coupled oscillator model,

$$\left(\begin{array}{cc}{E}_{c}\left(\theta \right) & \Omega /2\\ \Omega /2 & {E}_{{ex}}\end{array}\right)\left(\begin{array}{c}{\alpha }_{c}\\ {\alpha }_{{ex}}\end{array}\right)={E}_{{pol}}(\theta )\left(\begin{array}{c}{\alpha }_{c}\\ {\alpha }_{{ex}}\end{array}\right)$$

where *E*_ex_ is the energy of exciton, Ω the corresponding Rabi splitting energy. ${\left|{\alpha }_{c}\right|}^{2}$, ${\left|{\alpha }_{{ex}}\right|}^{2}$ are the Hopfield coefficients describing the photonic and excitonic weightings of the polaritons.

### Optical characterization

The real-space steady-state PL and reflectivity spectra were conducted in a confocal spectrometer (Horiba Evolution 800) by using a CW laser (532 nm). K-space energy-resolved reflectivity and PL mapping were measured in a home-built setup with the Fourier imaging configuration with a high numerical aperture 100× microscope objective (NA = 0.9). The PL mappings are excited by a 532 nm continuous-wave (CW) laser of ~100 μW. The emission from the microcavity was collected through the narrow entrance slit of the spectrometer (Horiba iHR550) and finally onto the 2D charge-coupled device (CCD) array (Horiba, Symphony II). In the *k*-space transient-reflectivity spectroscopy measurement, the excitation pulsed laser was taken from a Ti:Sapphire laser equipped with an ultrafast amplifier (Spectra-Physics) and a computer-controlled Optical Parametric Amplifier (1 kHz repetition rate, with a roughly 200 fs pulse width). The output beam was split into two paths. One beam excited the sample almost at normal incidence to serve as the pump beam. The second beam went through a mechanical delay stage (Newport, M-ILS 150CC DC Servo Linear Stage) and a sapphire crystal to generate a delayed continuum light to serve as the probe pulse. The spot size of the pump and probe beams was around 2 μm and 3 μm, respectively. PLE spectra were obtained with a supercontinuum light acting as the excitation source which is coupled to a monochromator. The excitation intensity was kept below 10 μW. A 655 nm long-pass filter was used to cut off the excitation photon that backscattered from the sample. All the measurements were carried out in a reflection configuration.

### Supplementary information

Supplementary Information

Peer Review File

Description of Additional Supplementary Files

supplementary movie 1

supplementary movie 2

## Data Availability

All data needed to evaluate the conclusions in the paper are present in the paper and/or the Supplementary Materials. Additional data related to this paper may be requested from the authors.
