# Supplementary material for: Energy transfer driven brightening of MoS2 by ultrafast polariton relaxation in microcavity MoS2/hBN/WS2 heterostructures
Source: Nat Commun. 2024 Feb 26;15:1747. doi: 10.1038/s41467-024-45554-y (PMC10897444; doi:10.1038/s41467-024-45554-y)
Supplement: Supplementary file 1 — Supplementary Information [file 41467_2024_45554_MOESM1_ESM.pdf]

Supplementary Information for

Energy transfer driven brightening of MoS_2 by ultrafast polariton relaxation in microcavity MoS_2/hBN/WS_2 heterostructures

Zehua Hu[1,12*], Tanjung Krisnanda[2,12], Antonio Fieramosca[2,12], Jiaxin Zhao[2], Qianlu Sun[1], Yuzhong Chen[3], Haiyun Liu[3], Yuan Luo[4], Rui Su[2], Junyong Wang[5], Kenji Watanabe[6], Takashi Taniguchi[7], Goki Eda[5], Xiao Renshaw Wang[2,8], Sanjib Ghosh[3], Kevin Dini[2*], Daniele Sanvitto[9], Timothy C. H. Liew[2], Qihua Xiong[3,4,10,11*]

[1]National Laboratory of Solid State Microstructures, School of Electronic Science and Engineering, and Collaborative Innovation Center of Advanced Microstructures, Nanjing University, Nanjing 210093, China.

[2]Division of Physics and Applied Physics, School of Physical and Mathematical Sciences, Nanyang Technological University 637371, Singapore, Singapore.

[3]Beijing Academy of Quantum Information Sciences, Beijing 100193, P.R. China

[4]State Key Laboratory of Low-Dimensional Quantum Physics, Department of Physics, Tsinghua University, Beijing 100084, P.R. China

[5]Department of Physics, National University of Singapore, Singapore 117542, Singapore

[6]Research Center for Functional Materials, National Institute for Materials Science, 1-1 Namiki, Tsukuba 305-0044, Japan

[7]International Center for Materials Nanoarchitectonics, National Institute for Materials Science, 1-1 Namiki, Tsukuba 305-0044, Japan

[8]School of Electrical and Electronic Engineering, Nanyang Technological University, 50 Nanyang Ave, 639798, Singapore

[9]CNR NANOTEC Institute of Nanotechnology, Lecce 73100, Italy

[10]Frontier Science Center for Quantum Information, Beijing 100084, P.R. China

[11]Collaborative Innovation Center of Quantum Matter, Beijing, P.R. China

[12]These authors contributed equally: Zehua Hu, Tanjung Krisnanda, Antonio Fieramosca

To whom correspondence should be addressed. Emails: qihua_xiong@tsinghua.edu.cn, kdini@ntu.edu.sg, zehuahu@nju.edu.cn

Supplementary part I: Sample fabrication

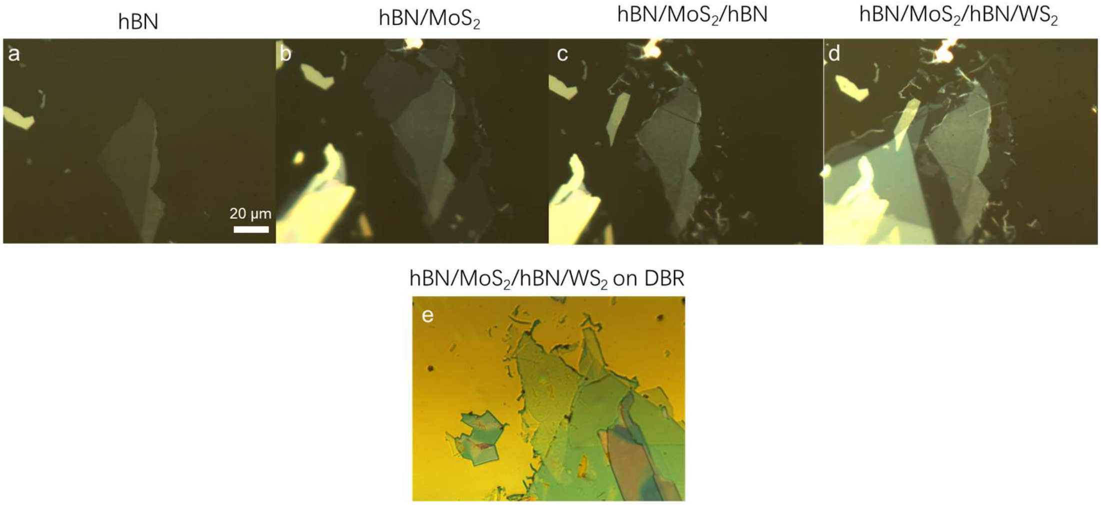

Supplementary Figure 1| Heterojunction fabrication process. (**a-d**) Optical image of the heterojunction made by the pick-up method. (**e**) Optical image of the heterojunction transferred onto the DBR substrate.

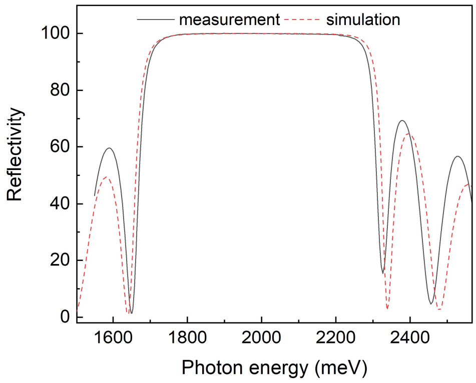

Supplementary Figure 2| Reflectivity spectrum of the DBR substrate.

Supplementary part II: Optical characterization

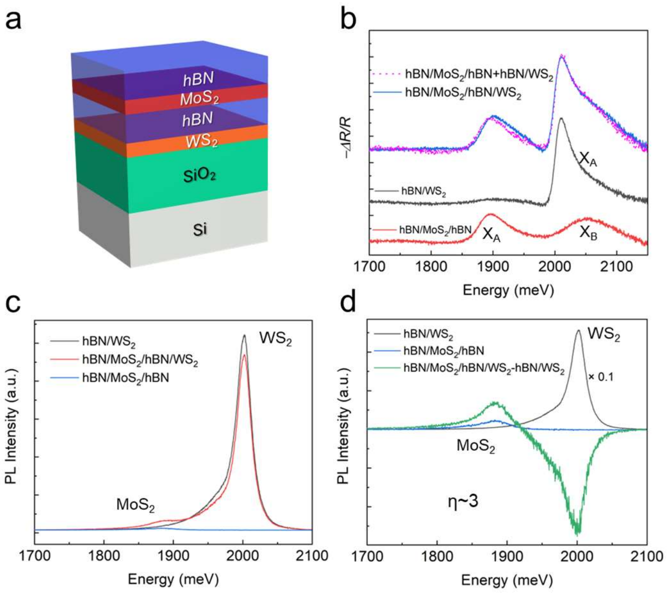

Supplementary Figure 3| FRET in het@SiO$_2$. (**a**) Schematic illustration of het@SiO$_2$. (**b, c**) Differential reflection (**b**) and PL (**c**) spectra in the different sample regions. (**d**) The comparison of PL spectra between hBN/WS$_2$ (black), hBN/MoS$_2$/hBN (blue) and hBN/MoS$_2$/hBN/WS$_2$ subtracting hBN/WS$_2$ (green). Note: PL spectra were obtained by the non-resonant excitation from a 532 nm continuous-wave (CW) laser of ~5 μW.

The intrinsic interlayer interaction in het@SiO$_2$ was learnt by comparing the differential reflection and PL spectra of hBN/MoS$_2$/hBN/WS$_2$, hBN/MoS$_2$/hBN, and hBN/WS$_2$ heterojunctions. The reflection spectrum of the hBN/MoS$_2$/hBN/WS$_2$ is almost the superposition of those of the hBN/MoS$_2$/hBN and hBN/WS$_2$, which indicates a weak interlayer coupling with a negligible transfer of dipole oscillator strength[1] (Supplementary Figure 3b). As known, for direct-bandgap semiconductor heterojunctions, the charge carrier transfer and exciton energy transfer are two dominant interlayer interactions. For the hBN/MoS$_2$/hBN/WS$_2$, the middle hBN layer of 2 nm almost entirely blocks the tunnelling current[2, 3], while FRET still exists for the space thickness (d) of <10 nm[2, 4]. Indeed, a FRET process is reflected by the weakened WS$_2$ PL intensity and strengthened MoS$_2$ PL intensity for the hBN/MoS$_2$/hBN/WS$_2$ in comparison with the corresponding intensity for hBN/MoS$_2$/hBN and hBN/WS$_2$ (Supplementary Figure 3c). By subtracting the spectrum of hBN/WS$_2$/hBN from that of hBN/MoS$_2$/hBN/WS$_2$, η is estimated to be ~3 (Supplementary Figure 3d), which is a typical value for FRET (Supplementary Table 2). Hence, without the cavity confinement, a typical FRET dominates the interlayer coupling in hBN/MoS$_2$/hBN/WS$_2$ heterojunction, which is also supported by the photoluminescence excitation (PLE) results (Supplementary Figure 6).

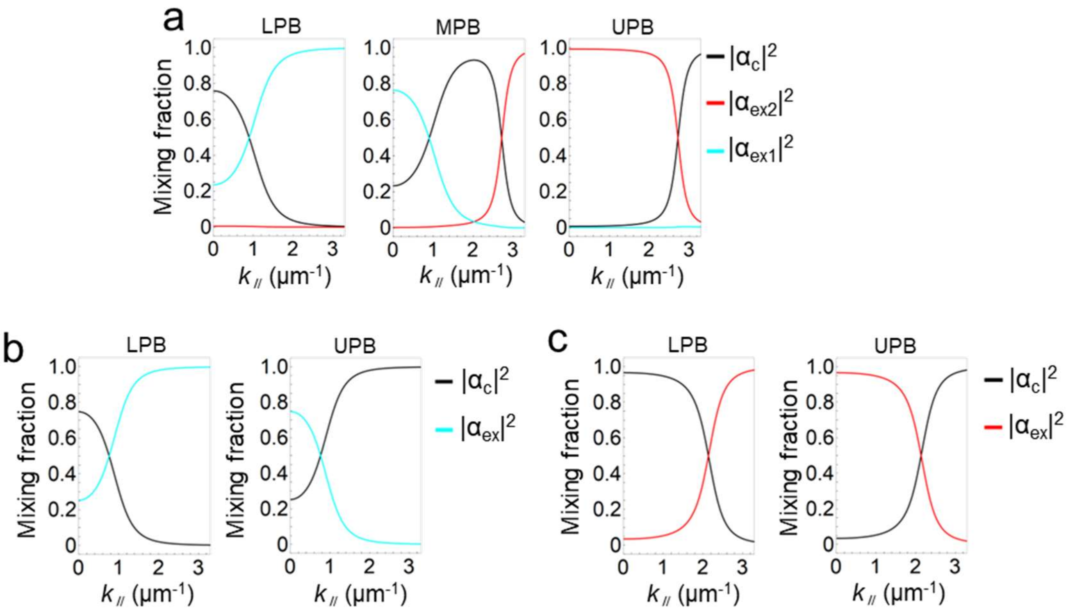

Supplementary Figure 4| Hopfield coefficient for het@cavity− (**a**), MoS_2@cavity (**b**) and WS_2@cavity (**c**).

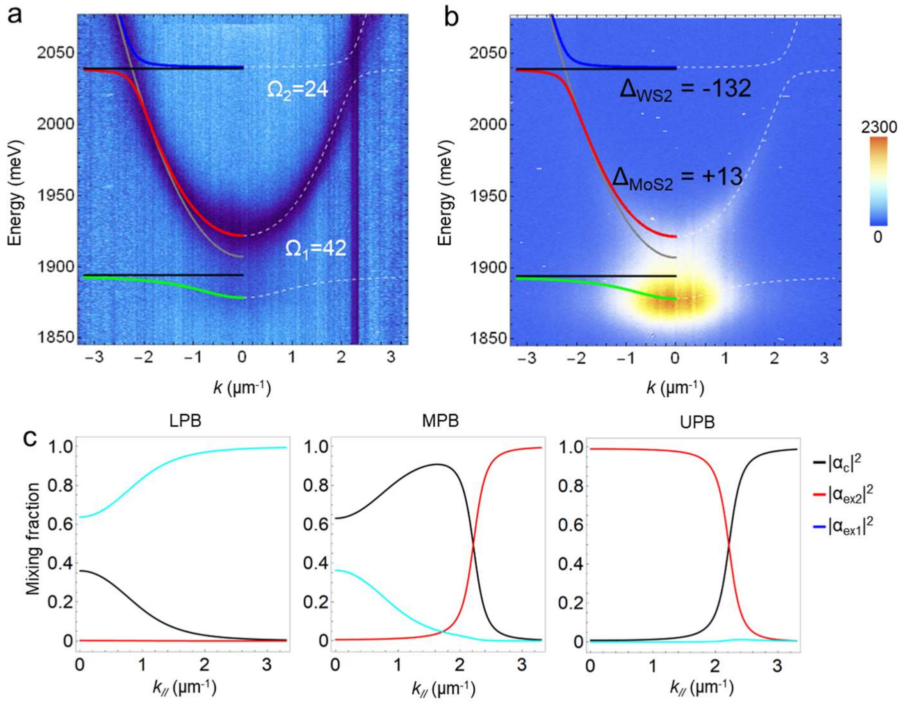

Supplementary Figure 5| Het@cavity+ with a positive detuning. (a, b) *k*-space energy-resolved reflectivity mapping (**a**), PL mapping (**b**). (**c**) Hopfield coefficient for LPB, MPB, and UPB, respectively.

The thickness of PMMA in het@cavity+ is slightly thinner than that in het@cavity−, leading to a blue-shift of the cavity photon mode.

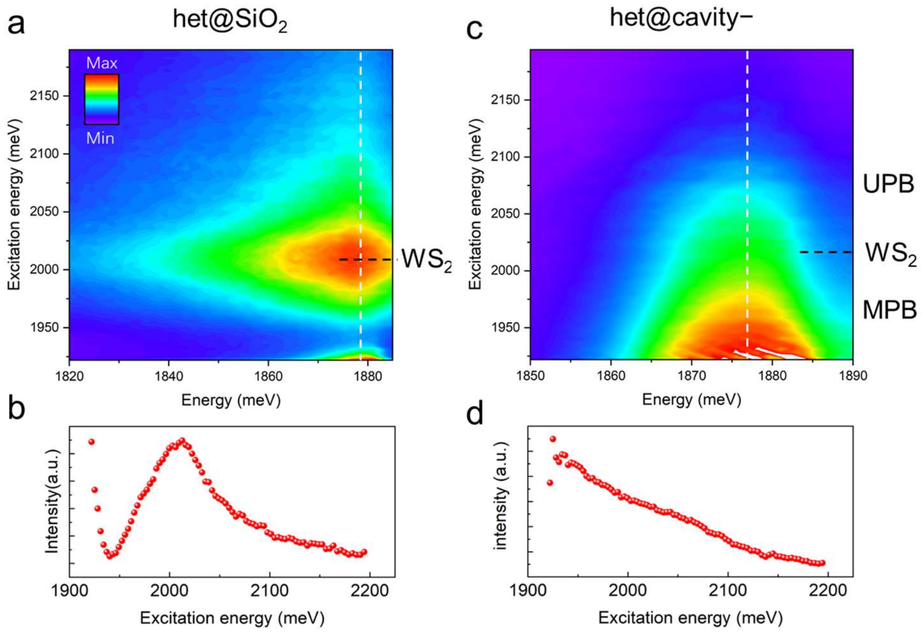

Supplementary Figure 6| Real-space PLE spectra. (**a, b**) PLE mapping (**a**), MoS_2 PL intensity with changing excitation energy (along the white dashed line in **a**) (**b**) of het@SiO_2. (**c, d**) PLE mapping (**c**), LPB PL intensity with changing excitation energy (along the white dashed line in **c**) (**d**) of het@cavity−.

In real-space PLE spectra, the MoS_2 emission of het@SiO_2 peaks at WS_2 exciton energy (Supplementary Figure 6a,b), demonstrating the FRET interaction, in agreement with the differential reflection and PL results in Supplementary Figure 3. On the contrary, the LPB emission of het@cavity− monotonously increases with decreasing the excitation energy to the LPB (Supplementary Figure 6c,d), which confirms the polariton relaxation mechanism.

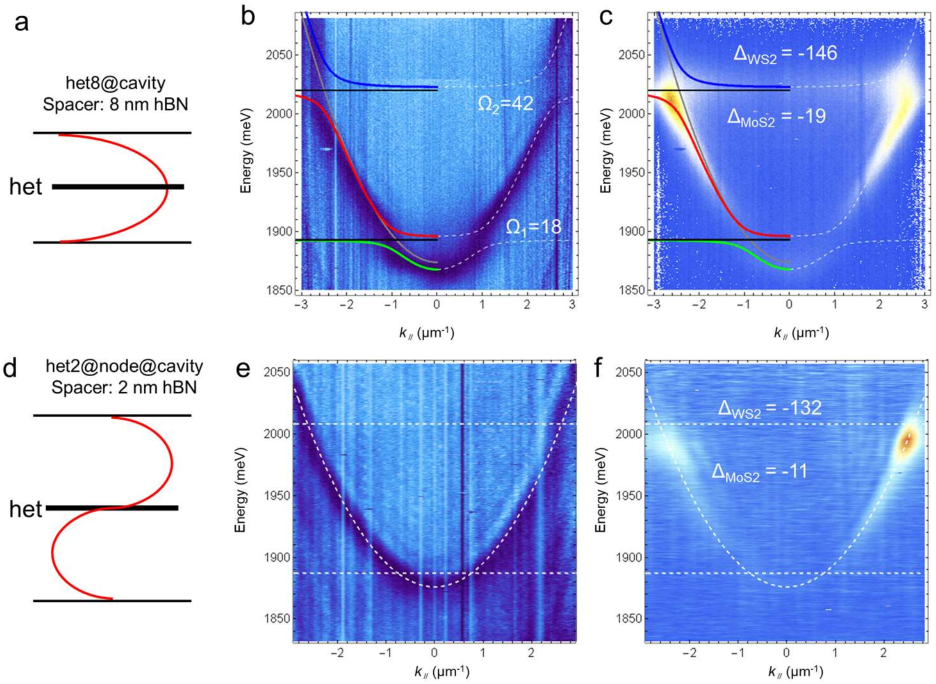

Supplementary Figure 7| (**a-c**) Schematic structure (**a**), *k*-space energy-resolved reflectivity mapping (**b**) and *k*-space energy-resolved PL mapping (**c**) of het8@cavity. (**d-f**) Schematic structure (**d**), *k*-space energy-resolved reflectivity mapping (**e**) and *k*-space energy-resolved PL mapping (**f**) of het2@node@cavity.

The heterojunction is located at the center of a half-cavity (λ/2 cavity) with an 8 nm hBN spacer in het8@cavity, *i.e.*, at the antinode of the electric field (thus reaching the strong coupling, Supplementary Figure 7a-c), while at the center of a full-cavity (λ cavity) with a 2 nm hBN spacer in het2@node@cavity, *i.e.*, at the node of the electric field (thus reaching the weak coupling, Supplementary Figure 7d-f).

het8@cavity shows the formation of three polariton branches with two anti-crossing points. The Rabi splittings are Ω_1=18 meV (MoS_2) and Ω_2=42 meV (WS_2), respectively (Supplementary Figure 7a-c), similar to the values of corresponding monolayer@cavity ($\Omega_1<\Omega_2$). The PL emission is obstructed by the bottleneck effect, with the clear signature of exciton reservoir emission (Supplementary Figure 7c). This result confirms that the exciton reservoir cannot assist the polariton relaxation.

het2@node@cavity features the perfect parabolic dispersion, demonstrating the weak coupling between exciton and photon (Supplementary Figure 7d-f). The PL emission from this contrast sample concentrates at the WS_2 exciton energy with negligible energy transfer to MoS_2 exciton (Supplementary Figure 7f), which confirms that FRET is not the dominant energy transfer mechanism in het@cavity- or het@cavity+. Therefore, the dominant mechanism is polariton-mediated energy transfer for het@cavity- or het@cavity+.

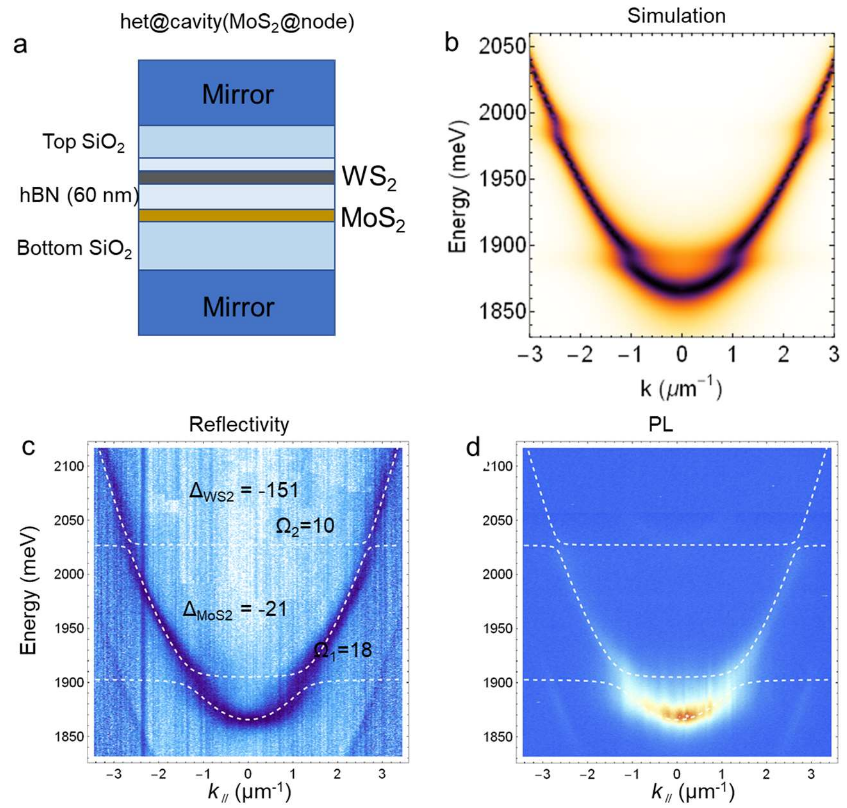

Supplementary Figure 8|(a-d) The schematic structure **(a)**, simulated reflectivity **(b)**, measured reflectivity **(c)**, and measured PL **(d)** of het60@cavity.

het60@cavity is separated by 60 nm hBN spacer, which completely quenches the direct FRET. Due to the different distribution of the photonic mode in the cavity, the light-matter coupling is tuned, with Ω_1=18 meV and Ω_2=10 meV, respectively, as seen from both theoretical and experimental results (Supplementary Figure 8 b,c). Ω_1 of het60@cavity equals to the Ω of MoS_2@cavity (18 meV). As a result, the *k*-resolved PL mostly assembles at the LPB, corresponding to a high polariton relaxation efficiency, which matches well with the calculated phase diagram (Figure 4a).

Therefore, all the experimental data support the theoretical prediction that the energy relaxation efficiency is closely related to the Rabi energies and therefore to the polariton relaxation.

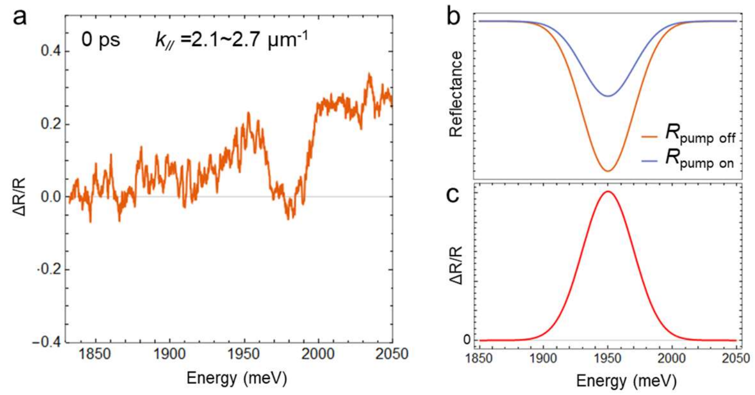

Supplementary Figure 9| (**a**) Integrated k-space transient-reflectivity spectrum of WS_2@cavity at 0 ps. (**b,c**) Schematic reflectance and corresponding $\Delta R/R$. Note: A simplified Gaussian function is used in (**b**), hence the simulated $\Delta R/R$ in (**c**) can only reflect the main feature of the polaritonic system.

The positive $\Delta R/R$ of WS_2@cavity at $k_{//}$=2.1~2.7 μm^{-1} in Supplementary Figure 9a indicates a pump-induced photobleaching process, as sketched in Supplementary Figure 9b,c. Specifically, the positive $\Delta R/R$ at 1950 and over 2000 meV corresponds to the photobleaching at LPB and UPB, respectively, while the dip at ~1970 meV to the negligible absorption at the Rabi splitting (Supplementary Figure 9a).

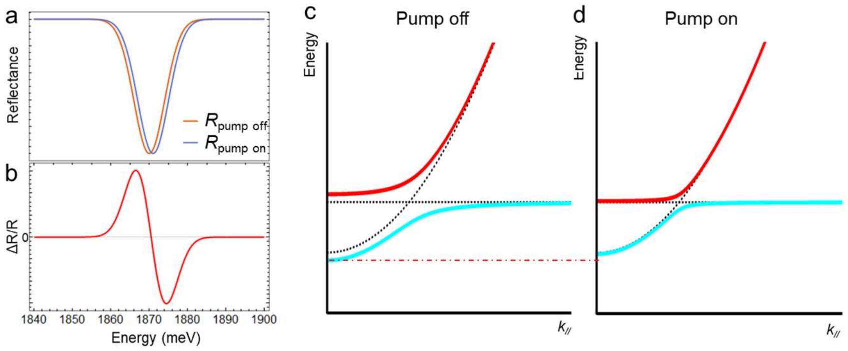

Supplementary Figure 10| The schematic illustration of a blue-shift in the *k*-space transient-reflectivity spectroscopy. (**a, b**) Blue-shift signal. (**c, d**) The dispersion with or without a pump.

The pump-induced blue shift shows a derivative signal in the integrated $\Delta R/R$ (Supplementary Figure 10a,b), which resembles the cases in Figure 3b,e. A blue shift in the polaritonic system represents a decrease of Rabi splitting energy (Ω), *i.e.,* the decrease of dipole oscillator strength, as schematically drawn in Supplementary Figure 10c,d.

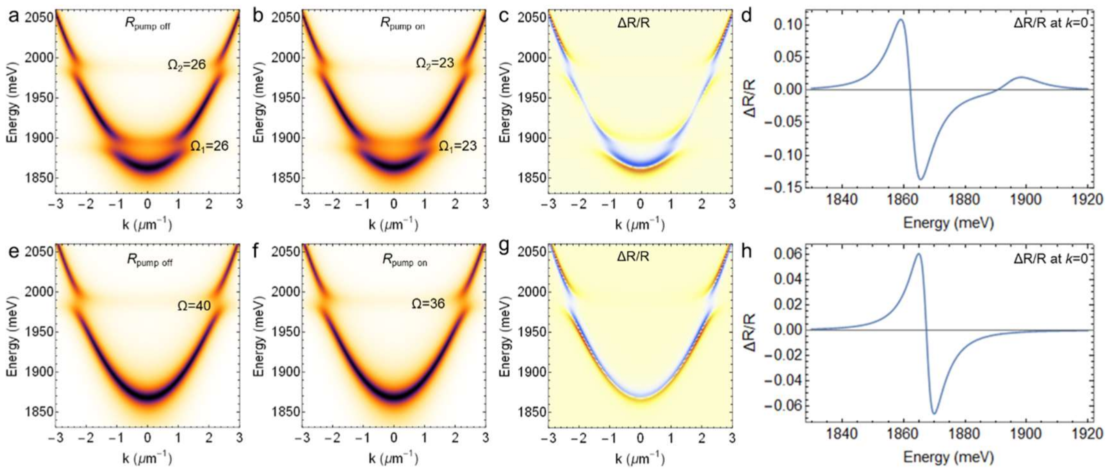

Supplementary Figure 11| Transfer matrix method simulation of the reflectivity spectroscopy. (**a-d**) k-space $R_{\text{pump off}}$ (**a**), k-space $R_{\text{pump on}}$ (**b**), k-space $\Delta R/R$ (**c**) and $\Delta R/R$ at k=0 (**d**) of Het@cavity-. (**e-h**) k-space $R_{\text{pump off}}$ (**e**), k-space $R_{\text{pump on}}$ (**f**), k-space $\Delta R/R$ (**g**) and $\Delta R/R$ at k=0 (**h**) of WS_2@cavity-.

Typically, under off-resonance pump excitation, the polariton relaxation towards the ground state of the dispersion is accompanied by a rigid blueshift of the LPB that is visible at every k in momentum space (broad in k). This energy shift is a result of either polariton-polariton interactions or interactions with the excitonic reservoir. The latter lasts longer (controlled by the exciton lifetime) and is not linked to the polariton lifetime. Actually, the polariton lifetime is mainly controlled by the quality factor of the microcavity (few picoseconds for our sample, see below). Therefore, after the fast polariton relaxation, the broad modulation visible at 50 ps is a consequence of residual interaction in the excitonic reservoir which shifts the energy of LPB. To better illustrate that a small energy shift of the LPB leads to a broad modulation in the differential pump-probe signal, we theoretically simulate the corresponding $\Delta R/R$ spectra by imposing a small quenching of the Rabi splitting. As shown in Supplementary Figure 11a-d, the Rabi energies are 26 and 26 meV for Ω_1 and Ω_2 without pump and decrease to 23 and 23 meV with pump. Although no obvious change can be found from the $R_{\text{pump off}}$ and $R_{\text{pump on}}$, the corresponding $\Delta R/R$ mapping shows a clear half-negative-half-positive signal in the LPB and the extracted $\Delta R/R$ spectrum at k=0 features the derivative signal. The simulated results are similar to the experimental results in Figure 3, supporting the blue-shift induced by the polariton-polariton repulsive interaction and residual interaction in the excitonic reservoir. WS_2@cavity also features similar results, as shown in Supplementary Figure 11e-h. Therefore, we interpret the broad modulation in momentum space at 50 ps as the result of residual interaction in the excitonic reservoir.

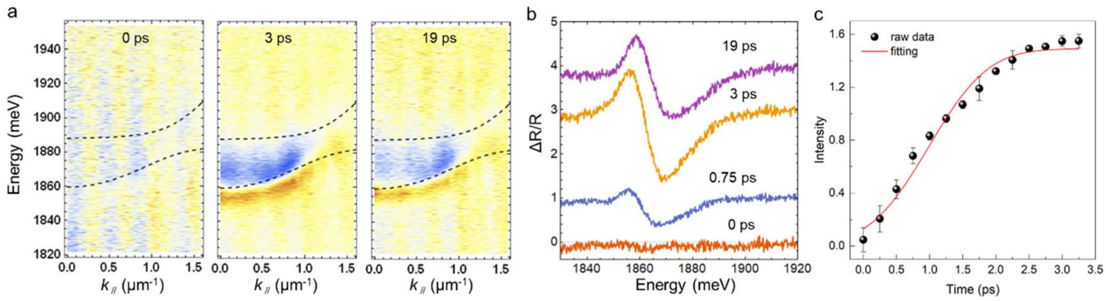

Supplementary Figure 12| (**a-c**) k-space transient-reflectivity spectroscopy mapping (**a**), the integrated $\Delta R/R$ in 0~0.33 μm^{-1} at typical time delays (**b**), and the intensity evolution of the derivative signal (**c**) of MoS_2@cavity. The standard error in c corresponds to the standard error of the data points.

The time-dependent ΔR/R spectra for MoS_2@cavity shows the clear derivative signal, corresponding to the decrease of Rabi energy induced by the polariton-polariton repulsive interaction. With the same fitting function, we get a rising time constant of 1.2±0.2 ps, which is similar to that of het@cavity- and shorter than that of WS_2@cavity. Here, the exciton-photon detuning is as small as ~-10 meV, which avoids the bottleneck effect. Therefore, the polariton relaxation rate is faster than that of WS_2@cavity.

Supplementary Note 1| The calculation of enhancement factor.

We calculated the enhancement factor by using the emission of het@cavity- with negligible WS_2's exciton contribution. Specifically, the LPB of the het@cavity- has negligible WS_2's exciton content (Supplementary Figure 4a). The lower limit of η is given by $\eta = I_{21}^{LPB}/I_1^{LPB+UPB} = 265$, where I_{21}^{LPB} and $I_1^{LPB+UPB}$ are the integrated polariton emission intensity from LPB of het@cavity- (red region) and LPB+UPB of MoS_2@cavity (black-outlined region) in real-space, respectively (Figure 2h). It should be emphasized that the polariton emission from the MPB (I_{21}^{MPB}) of het@cavity mainly comes from $|k_{//}|<1.6$ μm^{-1} with negligible E_{ex2} contribution (Supplementary Figure 4a). Therefore, the upper limit of η is given by $\eta = I_{21}^{LPB+MPB}/I_1^{LPB+UPB} = 441$.

Supplementary part III: Theory

Transfer-matrix (t-martrix) method

Following the well-known formalism[7-9], the T-matrix across a layer M in the TE polarization can be expressed as: $T_M = \begin{pmatrix} \cos(\alpha) & \frac{i}{q_M}\sin(\alpha) \\ iq_M\sin(\alpha) & \cos(\alpha) \end{pmatrix}$ where $\alpha = n_M(\omega)\frac{\omega}{c}L_M\cos(\theta_M)$ and $q_M = n_M(\omega)\frac{\mu_0}{c}$. We consider the thickness of each layer as: $L_{Ag} = 100\,nm$, $L_{TiO2} = 68.2\,nm$, $L_{SiO2} = 106.6\,nm$, $L_{\mathrm{hBN1}} = 2$ nm, $L_{\mathrm{hBN2}} = 11$ nm, $L_{SiO2\,Buffer} = 11$ nm, $L_{\mathrm{PMMA}} = 85$ nm, $L_{\mathrm{MoS2}} = 1$ nm, $L_{\mathrm{WS2}} = 1$ nm. The slight difference between the theoretical and experimental values is due to the error in the measurement of the thicknesses. Since the thicknesses of the mirror layers in the sample are not exactly the same, the theoretical model reaches a much higher quality factor for the same number of layers. To compensate for this we only consider two pairs of layers in the simulations. The refractive indices are taken from the literature[10-16].

The active layers (WS_2 and MoS_2 exciton states) are described in the framework of the T-matrix method, also following previously developed formalism[7-9, 17]. The monolayers associated with the T-matrix can be expressed as: $T_{QW} = \begin{pmatrix} 1 & 0 \\ \frac{2q_{QW}r_{QW}}{1+r_{QW}} & 1 \end{pmatrix}$, where q_{QW} is similar to q_M, although we assume the exciton resonance to be narrow enough such that the refractive index can be taken as a constant. The reflection coefficient can be derived as[17] $r_{QW} = i\Gamma_0/(\omega_0 - \omega - i(\Gamma_0 + \gamma))$ where Γ_0 is the exciton radiative broadening and $\hbar\omega_0$ is the resonance energy of the excitons. Considering these as fitting parameters, we get for the monolayers $\hbar\Gamma_{0,MoS2} = 0.05\,meV$, $\hbar\Gamma_{0,WS2} = 0.15\,meV$ and for the heterostructure $\hbar\Gamma_{0,MoS2} = 0.05\,meV$ $\hbar\Gamma_{0,WS2} = 0.13\,meV$. The exciton resonance energies are taken as: $\hbar\omega_{0,MoS2} = 1.885\,eV$, $\hbar\omega_{0,WS2} = 1.980\,eV$ for the monolayers and $\hbar\omega_{0,MoS2} = 1.885\,eV$, $\hbar\omega_{0,WS} = 2.020\,eV$ for the heterostructure. The decay rate of the exciton is taken identical for all excitons : $\gamma = 0.005\,meV$. The differences between the parameters in the monolayer structures and the heterostructure can be explained by disorder and strain distributions that change between different samples. One may note some discrepancies between the coupled oscillator model and the T-matrix formalism at high energy. This difference is due to the non-validity of the effective mass approximation for the cavity photon used in the coupled oscillator model. Indeed this approximation requires working close to the lowest energy of the optical mode. Comparing directly the T-matrix simulations and the experimental data, we conclude to good agreement between the experiment and the theory.

The coupled rate equations

Here we construct the coupled rate equations for the polariton populations in different branches, leading to the theoretical model for the PL observed in experiments, as shown in Figure 2b,d,f in the main text. Rate equation is a tool that has been useful for describing the dynamics of the population of polaritons or excitons[18-21]. We will present the case for het@cavity− (involving three polariton branches) explicitly, while the ones for WS_2@cavity and MoS_2@cavity configurations follow as special cases. First, we consider the polariton population for all the branches (UPB, MPB, and LPB) in k-space and its evolution to the steady-state regime. The photonic part of these values corresponds to the observed PL, which with broadening in energy gives the simulated PL in the left half of Figure 2b (also Figure 2d,f for the case of MoS_2@cavity and WS_2@cavity, respectively).

The coupled rate equations for the polariton populations read

$$\frac{dN_j^U}{dt} = \left(P_1 X_{1,j}^U + P_2 X_{2,j}^U - \gamma C_j^U\right) N_j^U + S^{UU} + S^{UM} + S^{UL},$$

$$\frac{dN_j^M}{dt} = \left(P_1 X_{1,j}^M + P_2 X_{2,j}^M - \gamma C_j^M\right) N_j^M + S^{MM} + S^{MU} + S^{ML},$$

$$\frac{dN_j^L}{dt} = \left(P_1 X_{1,j}^L + P_2 X_{2,j}^L - \gamma C_j^L\right) N_j^L + S^{LL} + S^{LU} + S^{LM}, \qquad (1)$$

where the superscripts $\{U, M, L\}$ respectively denote the UPB, MPB, and LPB, while the subscript j indicates the corresponding label for the point in k-space (uniform in k). The exciton fractions, denoted by $X_{\{1,2\},j}^{\{U,M,L\}}$, are obtained from the coupled oscillator model (Hopfield coefficients in Supplementary Figure 4), where 1 (2) indicates excitons in MoS_2 (WS_2). $C_j^{\{U,M,L\}}$ denotes the corresponding photonic fraction, $P_{1\,(2)}$ the effective pump for the excitons in MoS_2 (WS_2), and γ the decay rate for the photonic part of the polariton. The scattering terms originate from phonon-mediated exciton scattering[22, 23], giving rise to both intra-branch (S^{UU}, S^{MM}, and S^{LL}) and inter-branch (S^{UM}, S^{UL}, S^{MU}, S^{ML}, S^{LU}, and S^{LM}) scattering. The explicit expressions are written as

$$S^{UU} = \sum_{k,\,E_j^U < E_k^U} \left(W_1 X_{1,j}^U X_{1,k}^U + W_2 X_{2,j}^U X_{2,k}^U\right)\left|E_j^U - E_k^U\right| \begin{pmatrix} N_k^U\left(N_j^U + 1\right)\left(n_{ph}\left(\left|E_j^U - E_k^U\right|\right) + 1\right) \\ -N_j^U\left(N_k^U + 1\right) n_{ph}\left(\left|E_j^U - E_k^U\right|\right) \end{pmatrix}$$
$$+ \sum_{k,\,E_j^U > E_k^U} \left(W_1 X_{1,j}^U X_{1,k}^U + W_2 X_{2,j}^U X_{2,k}^U\right)\left|E_j^U - E_k^U\right| \begin{pmatrix} N_k^U\left(N_j^U + 1\right) n_{ph}\left(\left|E_j^U - E_k^U\right|\right) \\ -N_j^U\left(N_k^U + 1\right)\left(n_{ph}\left(\left|E_j^U - E_k^U\right|\right) + 1\right) \end{pmatrix},$$

$$S^{UM} = \sum_{k,\,E_j^U > E_k^M} \left(W_1 X_{1,j}^U X_{1,k}^M + W_2 X_{2,j}^U X_{2,k}^M\right)\left|E_j^U - E_k^M\right| \begin{pmatrix} N_k^M\left(N_j^U + 1\right) n_{ph}\left(\left|E_j^U - E_k^M\right|\right) \\ -N_j^U\left(N_k^M + 1\right)\left(n_{ph}\left(\left|E_j^U - E_k^M\right|\right) + 1\right) \end{pmatrix},$$

$$S^{UL} = \sum_{k,\,E_j^U > E_k^L} \left(W_1 X_{1,j}^U X_{1,k}^L + W_2 X_{2,j}^U X_{2,k}^L\right)\left|E_j^U - E_k^L\right| \begin{pmatrix} N_k^L\left(N_j^U + 1\right) n_{ph}\left(\left|E_j^U - E_k^L\right|\right) \\ -N_j^U\left(N_k^L + 1\right)\left(n_{ph}\left(\left|E_j^U - E_k^L\right|\right) + 1\right) \end{pmatrix},$$

$$S^{MM} = \sum_{k,\,E_j^M < E_k^M} \left(W_1 X_{1,j}^M X_{1,k}^M + W_2 X_{2,j}^M X_{2,k}^M\right)\left|E_j^M - E_k^M\right| \begin{pmatrix} N_k^M\left(N_j^M + 1\right)\left(n_{ph}\left(\left|E_j^M - E_k^M\right|\right) + 1\right) \\ -N_j^M\left(N_k^M + 1\right) n_{ph}\left(\left|E_j^M - E_k^M\right|\right) \end{pmatrix}$$
$$+ \sum_{k,\,E_j^M > E_k^M} \left(W_1 X_{1,j}^M X_{1,k}^M + W_2 X_{2,j}^M X_{2,k}^M\right)\left|E_j^M - E_k^M\right| \begin{pmatrix} N_k^M\left(N_j^M + 1\right) n_{ph}\left(\left|E_j^M - E_k^M\right|\right) \\ -N_j^M\left(N_k^M + 1\right)\left(n_{ph}\left(\left|E_j^M - E_k^M\right|\right) + 1\right) \end{pmatrix},$$

$$S^{MU} = \sum_{k,\,E_j^M<E_k^U} \left(W_1 X_{1,j}^M X_{1,k}^U + W_2 X_{2,j}^M X_{2,k}^U\right)\left|E_j^M - E_k^U\right| \begin{pmatrix} N_k^U\left(N_j^M+1\right)\left(n_{ph}\left(\left|E_j^M - E_k^U\right|\right)+1\right) \\ -N_j^M\left(N_k^U+1\right)n_{ph}\left(\left|E_j^M - E_k^U\right|\right) \end{pmatrix},$$

$$S^{ML} = \sum_{k,\,E_j^M>E_k^L} \left(W_1 X_{1,j}^M X_{1,k}^L + W_2 X_{2,j}^M X_{2,k}^L\right)\left|E_j^M - E_k^L\right| \begin{pmatrix} N_k^L\left(N_j^M+1\right)n_{ph}\left(\left|E_j^M - E_k^L\right|\right) \\ -N_j^M\left(N_k^L+1\right)\left(n_{ph}\left(\left|E_j^M - E_k^L\right|\right)+1\right) \end{pmatrix},$$

$$S^{LL} = \sum_{k,\,E_j^L<E_k^L} \left(W_1 X_{1,j}^L X_{1,k}^L + W_2 X_{2,j}^L X_{2,k}^L\right)\left|E_j^L - E_k^L\right| \begin{pmatrix} N_k^L\left(N_j^L+1\right)\left(n_{ph}\left(\left|E_j^L - E_k^L\right|\right)+1\right) \\ -N_j^L\left(N_k^L+1\right)n_{ph}\left(\left|E_j^L - E_k^L\right|\right) \end{pmatrix}$$

$$+\sum_{k,\,E_j^L>E_k^L} \left(W_1 X_{1,j}^L X_{1,k}^L + W_2 X_{2,j}^L X_{2,k}^L\right)\left|E_j^L - E_k^L\right| \begin{pmatrix} N_k^L\left(N_j^L+1\right)n_{ph}\left(\left|E_j^L - E_k^L\right|\right) \\ -N_j^L\left(N_k^L+1\right)\left(n_{ph}\left(\left|E_j^L - E_k^L\right|\right)+1\right) \end{pmatrix},$$

$$S^{LU} = \sum_{k,\,E_j^L<E_k^U} \left(W_1 X_{1,j}^L X_{1,k}^U + W_2 X_{2,j}^L X_{2,k}^U\right)\left|E_j^L - E_k^U\right| \begin{pmatrix} N_k^U\left(N_j^L+1\right)\left(n_{ph}\left(\left|E_j^L - E_k^U\right|\right)+1\right) \\ -N_j^L\left(N_k^U+1\right)n_{ph}\left(\left|E_j^L - E_k^U\right|\right) \end{pmatrix},$$

$$S^{LM} = \sum_{k,\,E_j^L<E_k^M} \left(W_1 X_{1,j}^L X_{1,k}^M + W_2 X_{2,j}^L X_{2,k}^M\right)\left|E_j^L - E_k^M\right| \begin{pmatrix} N_k^M\left(N_j^L+1\right)\left(n_{ph}\left(\left|E_j^L - E_k^M\right|\right)+1\right) \\ -N_j^L\left(N_k^M+1\right)n_{ph}\left(\left|E_j^L - E_k^M\right|\right) \end{pmatrix}, \quad (2)$$

where $n_{ph}(x)$ stands for phonon distribution as detailed in the main text, $W_{1\,(2)}$ is the exciton scattering coefficient. We note that a scattering process from a higher to lower energy emits a phonon and that of the reverse requires the absorption of one, hence the factor $n_{ph}(x)+1$ and $n_{ph}(x)$, respectively. This in turn guarantees that the whole system undergoes energy relaxation. The energy of the polariton $E_j^{\{U,M,L\}}$ (in meV) corresponds to the jth point in k-space, which is obtained from the coupled oscillator model.

In simulations, we take $(P_1, P_2, W_1, W_2)/\gamma$ as fitting parameters. The initial values for the population are taken as random, *i.e.*, $N_j^{\{U,M,L\}}(0) \in [0,1]$. After the evolution, one gets the steady-state values (at $\gamma t = \tau$) for the population, $N_j^{\{U,M,L\}}(\tau)$. The photonic part $n_j^{\{U,M,L\}} \equiv N_j^{\{U,M,L\}}(\tau) C_j^{\{U,M,L\}}$ is responsible for the observed PL. The broadening in energy is incorporated as follows. For the jth point in the k-space, the energy dependence follows a Gaussian broadening:

$$n_j^{\{U,M,L\}}(E) = n_j^{\{U,M,L\}} \frac{1}{\sigma_j^{\{U,M,L\}}\sqrt{2\pi}} \exp\left(-\frac{\left(E - E_j^{\{U,M,L\}}\right)^2}{2\left(\sigma_j^{\{U,M,L\}}\right)^2}\right), \quad (3)$$

where the width $\sigma_j^{\{U,M,L\}} = X_{1,j}^{\{U,M,L\}}\Gamma_1 + X_{2,j}^{\{U,M,L\}}\Gamma_2 + C_j^{\{U,M,L\}}\Gamma$ with Γ_1, Γ_2, and Γ being the width of the MoS_2 excitons, WS_2 excitons, and cavity photons, respectively. The exciton widths are fixed at $\Gamma_1 = \Gamma_2 = 1$ meV, while Γ may vary for different configurations. The simulated PL for the jth point is given by $n_{PL,j}(E) = n_j^U(E) + n_j^M(E) + n_j^L(E)$. We have also taken into account a small shift and noises in measurements, *i.e.*, $n_{PL,j}(E) \to n_{PL,j}(E) + n_0 + [0, \epsilon_j(E)]$, where n_0 is a small constant and the last term is a random number between 0 and $\epsilon_j(E)$ generated for every j and E.

The configurations of MoS_2 and WS_2 are special cases of the method described above, where there are only two polariton branches and one type of excitons considered in each case. For the simulated PL in Figure 2d,f in the main text, the fitted parameters are summarized in Table 3. The total number of points in the k-space for each polariton branch is 50.

In the simulation of Figure 4, we fix the fitting parameters as used in the simulation of Figure 2b except for Ω_1 and Ω_2.

Supplementary Table 1| The detailed thickness information of all the constitutive layers.

	Layer	Thickness
	Ag	100 nm
	PMMA	90 nm
	hBN	15 nm
	MoS_2	1 nm
	hBN	2 nm
	WS_2	1 nm
	SiO_2	100 nm
6 pairs	TiO_2	68.2 nm
	SiO_2	106.6 nm

The same fabrication methods are used for MoS_2@cavity and WS_2@cavity. The hBN and PMMA thicknesses are finely tuned to ensure a similar cavity cut off energy to that in het@cavity−.

Supplementary Table 2| Summarized energy transfer time constant and enhancement factor.

Heterojunction	Donor-acceptor distance/mechanism	Energy transfer characteristic time	Enhancement factor (η)	Reference
**WS_2/hBN/MoS_2 in FP cavity**	**3 nm/Polariton relaxation**	**1.3 ps**	**440**	**This study**
**WS_2/hBN/MoS_2 on SiO_2**	3 nm/FRET	none	3	
**Single-wall carbon nanotube bundles**	0.34 nm/FRET	1.8 ps	none	24
**CdSe quantum dot-MoS_2**	6.3 nm/FRET	4 ns	3.3	25
**Molecules−graphene**	5 nm/FRET	39 ps	none	26
**WS_2/$MoSe_2$**	1 nm/FRET	1 ps	<1	4
**WS_2/hBN/$MoSe_2$**	3 nm/FRET	38 ps	2	2
**WSe_2/$MoTe_2$**	1 nm/DET	0.2 ps	2	27
**Pentancene/$MoSe_2$**	1 nm/DET	none	2	28
**2D perovskite/WS_2**	1 nm/FRET	3 ns	8	29
**J-aggregates in FP cavity**	<1 nm/Polariton relaxation	1.5 ps	none	30
**J-aggregates in FP cavity**	100 nm/Polariton relaxation	25 ps	none	31

Supplementary Table 3| Summary of parameters used in simulations for Figure 2 b,d,f in the main text.

Configuration	P_1/γ	P_2/γ	W_1/γ	W_2/γ	Γ [meV]
**het@cavity−**	0.64	1.28	0.030	0.004	11.5
**MoS_2@cavity**	0.59	-	0.030	-	14.0
**WS_2cavity**	-	2.2	-	0.004	10.0

References

1. Valeur, B. & Berberan-Santos, M.N. Molecular fluorescence: principles and applications. (John Wiley & Sons, 2012).
2. Hu, Z. et al. Trion-mediated Forster resonance energy transfer and optical gating effect in WS2/hBN/MoSe2 heterojunction. *ACS Nano* **14**, 13470-13477 (2020).
3. Liu, X. et al. Manipulating charge and energy transfer between 2D Atomic layers via heterostructure engineering. *Nano Lett.* **20**, 5359-5366 (2020).
4. Kozawa, D. et al. Evidence for fast interlayer energy transfer in $MoSe_2$/WS_2 heterostructures. *Nano Lett.* **16**, 4087-4093 (2016).
5. Lyo, S.K. Energy transfer from an electron-hole plasma layer to a quantum well in semiconductor structures. *Phys. Rev. B* **81** (2010).
6. Wurdack, M. et al. Enhancing Ground-State Population and Macroscopic Coherence of Room-Temperature WS_2 Polaritons through Engineered Confinement. *Phys. Rev. Lett.* **129**, 147402 (2022).

7. Vladimirova, M., Kavokin, A. & Kaliteevski, M. Dispersion of bulk exciton polaritons in a semiconductor microcavity. *Phys. Rev. B* **54**, 14566 (1996).
8. Kavokin, A.V., Baumberg, J.J., Malpuech, G. & Laussy, F.P. Microcavities, Vol. 21. (Oxford university press, 2017).
9. Krisnanda, T. et al. Room temperature light-mediated long-range coupling of excitons in perovskites. *Adv. Opt. Mater.* **9**, 2001835 (2021).
10. DeVore, J.R. Refractive indices of rutile and sphalerite. *J. Opt. Soc. Am.* **41**, 416-419 (1951).
11. Malitson, I.H. Interspecimen comparison of the refractive index of fused silica. *J. Opt. Soc. Am.* **55**, 1205-1209 (1965).
12. Beadie, G., Brindza, M., Flynn, R.A., Rosenberg, A. & Shirk, J.S. Refractive index measurements of poly (methyl methacrylate)(PMMA) from 0.4–1.6 μm. *Appl. Opt.* **54**, F139-F143 (2015).
13. Lee, S.Y., Jeong, T.Y., Jung, S. & Yee, K.J. Refractive index dispersion of hexagonal boron nitride in the visible and near-Infrared. *Phys. Status Solidi* **256**, 1800417 (2019).
14. Hsu, C. et al. Thickness-dependent refractive index of 1L, 2L, and 3L MoS2, MoSe2, WS2, and WSe2. *Adv. Opt. Mater.* **7**, 1900239 (2019).
15. Song, B. et al. Layer-dependent dielectric function of wafer-scale 2D MoS2. *Adv. Optical Mater.* **7**, 1801250 (2019).
16. Jiang, Y., Pillai, S. & Green, M.A. Realistic silver optical constants for plasmonics. *Sci. Rep.* **6**, 1-7 (2016).
17. Vladimirova, M., Ivchenko, E. & Kavokin, A. Exciton polaritons in long-period quantum-well structures. *Semiconductors* **32**, 90-95 (1998).
18. Amthor, M. et al. Optical bistability in electrically driven polariton condensates. *Phys. Rev. B* **91**, 081404 (2015).
19. Solnyshkov, D., Terças, H., Dini, K. & Malpuech, G. Hybrid Boltzmann–Gross-Pitaevskii theory of Bose-Einstein condensation and superfluidity in open driven-dissipative systems. *Phys. Rev. A* **89**, 033626 (2014).
20. Lyons, T. et al. Giant effective Zeeman splitting in a monolayer semiconductor realized by spin-selective strong light-matter coupling. *arXiv preprint arXiv:.05859* (2021).
21. Zhang, Q. et al. Electric Field Modulation of 2D Perovskite Excitonics. *J. Phys. Chem. Lett.* **13**, 7161-7169 (2022).
22. Tassone, F., Piermarocchi, C., Savona, V., Quattropani, A. & Schwendimann, P. Bottleneck effects in the relaxation and photoluminescence of microcavity polaritons. *Phys. Rev. B* **56**, 7554 (1997).
23. Tassone, F. & Yamamoto, Y. Exciton-exciton scattering dynamics in a semiconductor microcavity and stimulated scattering into polaritons. *Phys. Rev. B* **59**, 10830 (1999).
24. Koyama, T. et al. Ultrafast exciton energy transfer in bundles of single-walled carbon nanotubes. *J. Phys. Chem. Lett.* **2**, 127-132 (2011).
25. Prins, F., Goodman, A.J. & Tisdale, W.A. Reduced dielectric screening and enhanced energy transfer in single- and few-layer MoS_2. *Nano. Lett.* **14**, 6087-6091 (2014).
26. Gaudreau, L. et al. Universal distance-scaling of nonradiative energy transfer to graphene. *Nano Lett.* **13**, 2030-2035 (2013).
27. Wu, L., Chen, Y., Zhou, H. & Zhu, H. Ultrafast energy transfer of both bright and dark

excitons in 2D van der Waals heterostructures beyond dipolar coupling. *ACS Nano* **13**, 2341-2348 (2019).

28. Cheng, C.H., Li, Z., Hambarde, A. & Deotare, P.B. Efficient energy transfer across organic-2D inorganic heterointerfaces. *ACS Appl. Mater. Interfaces* **10**, 39336-39342 (2018).
29. Zhang, Q., Linardy, E., Wang, X. & Eda, G. Excitonic energy transfer in heterostructures of quasi-2D perovskite and monolayer WS2. *ACS Nano* **14**, 11482-11489 (2020).
30. Zhong, X. et al. Non-radiative energy transfer mediated by hybrid light-matter states. *Angew. Chem. Int. Ed.* **55**, 6202-6206 (2016).
31. Zhong, X. et al. Energy transfer between spatially separated entangled molecules. *Angew. Chem. Int. Ed.* **56**, 9034-9038 (2017).
