# Supplementary material for: Energy transfer driven brightening of MoS2 by ultrafast polariton relaxation in microcavity MoS2/hBN/WS2 heterostructures
Source: Nat Commun. 2024 Feb 26;15:1747. doi: 10.1038/s41467-024-45554-y (PMC10897444; doi:10.1038/s41467-024-45554-y)
Supplement: Supplementary file 3 — Description of Additional Supplementary Files [file 41467_2024_45554_MOESM3_ESM.pdf]

Supplementary Movie 1

Description: *K*-space transient reflectivity of WS_2@cavity.

Supplementary Movie 2

Description: *K*-space transient reflectivity of het@cavity-.
