# Supplementary figures and images for: Energy transfer driven brightening of MoS2 by ultrafast polariton relaxation in microcavity MoS2/hBN/WS2 heterostructures
Source: Nat Commun. 2024 Feb 26;15:1747. doi: 10.1038/s41467-024-45554-y (PMC10897444; doi:10.1038/s41467-024-45554-y)

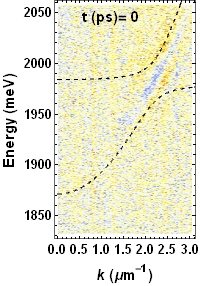

Supplement: Supplementary file 4 — supplementary movie 1 [file 41467_2024_45554_MOESM4_ESM.gif]

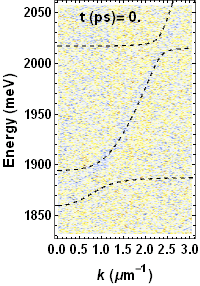

Supplement: Supplementary file 5 — supplementary movie 2 [file 41467_2024_45554_MOESM5_ESM.gif]
